# Risk factor assessment and microbiome analysis in peritoneal dialysis-related peritonitis reveal etiological characteristics

**DOI:** 10.3389/fimmu.2024.1443468

**Published:** 2024-11-14

**Authors:** Li Zhang, Hongrui Zhang, Sensen Su, Ye Jia, Chenyang Liang, Yuan Fang, Dengwei Hong, Tianyu Li, Fuzhe Ma

**Affiliations:** ^1^ Department of Nephrology, The First Hospital of Jilin University, Changchun, China; ^2^ Genoxor Medical Science and Technology Inc., Shanghai, China

**Keywords:** peritoneal dialysis-related peritonitis, risk factor, mNGS, pathogenic identification, gut microbiome

## Abstract

**Background:**

Peritoneal dialysis-related peritonitis (PDRP) is one of the most common complications of peritoneal dialysis (PD). Understanding the risk factors and etiological characteristics is indispensable for infection prevention and improving the outcome and life quality.

**Methods:**

A total of 70 PD patients were separated into the PDRP group (n=25) and the control group (n=45). Variables, including gender, age, body mass index, primary diseases, and history of basic diseases, in the two groups were analyzed to assess the risk factors of PDRP. Metagenomic next-generation sequencing (mNGS) and microbial culture were compared in detecting pathogenic microorganisms. Gut microbiota analysis was performed in 35 PDRP patients based on mNGS data.

**Results:**

Dialysis time and times of dialysate change were the risk factors of PDRP, and times of dialysate change was the independent risk factor of PDRP (p = 0.046). mNGS produced higher sensitivity (65.79%) than microbial culture (36.84%) in identifying pathogenic microorganisms. *Staphylococcus aureus* and *Klebsiella pneumoniae* (four cases) were the most frequent pathogens causing PDRP, followed by *Staphylococcus capitis* (three cases). β diversity of the gut microbiota was significantly different between patients with fewer times of dialysate change (≤4) and more (>5), as well as between patients with gram-positive (G+) bacterial and gram-negative (G−) bacterial infection.

**Conclusion:**

The dialysis time and times of dialysate changes not only are risk factors for peritonitis in PD patients but also stimulate significant changes in the gut microbiome structure in PDRP patients. These findings may provide a novel viewpoint for the management of patients with PDRP.

## Introduction

Peritoneal dialysis (PD) is an essential and dominant renal replacement therapeutic strategy for end-stage renal disease (ESRD) (1). Approximately 11% of ESRD patients choose PD as a kidney replacement treatment, and this number may vary by country and region (2, 3). Compared with hemodialysis, PD has the following advantages, including fewer hospital visits, less occurrence of hypotension, less impact on the cardiovascular system, no need for anticoagulants, better preservation of residual kidney function (4, 5), a more independent lifestyle, and greater affordability (6). With the advances in dialysis pipelines, dialysate, and dialysis technology, complications of PD have decreased so far (1, 7, 8). However, the morbidity of peritoneal dialysis-related peritonitis (PDRP) is stubbornly high and is the leading cause of dialysis withdrawal and death (9). Several risk factors of PDRP have been certified. Some non-modifiable variables include age, female, black ethnicity, low socioeconomic status, diabetes, and cardiovascular disease (10, 11). However, no consensus has been reached on the risk factors of PDRP. Exploration of PDRP risk factors is indispensable to provide critical guidelines for reducing PDRP rates and improving PD patients’ prognoses.

Metagenomic next-generation sequencing (mNGS) is an emerging tool for identifying microbial DNA and/or RNA in clinical specimens and is changing the diagnosis landscape of infectious diseases (12). This technology allows for identification and genomic characterization of bacteria, fungi, parasites, and viruses directly without the need for *a priori* knowledge of a specific pathogen (13). So far, mNGS has been applied for detection of pathogens in dialysis effluent of PDRP, with a significantly higher detection rate than traditional culture (14). Meanwhile, mNGS is prior to microbial culture and especially recommended in PDRP patients who previously received antibiotics (15). In order to explore approaches to improve the diagnostic rate of mNGS in detecting peritonitis pathogens, we assessed the detection performance of mNGS by comparing the sensitivity and specificity with those of microbial culture in dialysis effluent in this study.

In healthy individuals, numerous microbial populations are located in diverse body parts to maintain homeostasis in the immune and metabolism (16). Nevertheless, plenty of diseases are closely associated with dysbacteriosis. Disturbance in gut microbiota may result in intestinal dysbiosis, intestinal barrier dysfunction, and bacterial translocation (17). It has been reported that patients undergoing PD therapy presented lower species richness in the peritoneal microbiome (18). Moreover, gut microbial metabolite has been proven to increase peritoneal inflammation and peritonitis risk in PD (19). Therefore, gut microbiome analysis in PDRP patients is indispensable to determine microbial characteristics, which may reveal more risk factors of peritonitis in this cohort of patients.

Herein, we analyzed the potential risk factors of PDRP and investigated the gut microbiome features in PD based on the mNGS approach. This study aimed to provide innovative proofs and references for preventing peritonitis and treating PD patients.

## Materials and methods

### Study population

From 01/08/2015 to 31/08/2022, patients who received PD therapy for over 2 months in The First Hospital of Jilin University were recruited for this study. The exclusion criteria were as follows: 1) diagnosis of peritonitis within 2 months after PD initiation; 2) other infection with peritonitis; 3) presence of malignancy; 4) immunosuppressive cases; 5) liver cirrhosis and hepatitis and other systemic diseases; 6) use probiotics, antibiotics, prebiotics, and other drugs within 2 weeks. A total of 90 PD patients were screened, but only 70 with complete clinical data were included in the further analysis. This study was conducted in accordance with the Declaration of Helsinki and was approved by the ethical committee of the First Hospital of Jilin University (No. 21K026-001). Informed consent was obtained from all patients who participated in this study.

### Demographic and clinical data

For these 70 patients with full clinical data, the baseline information (including gender, age, body mass index (BMI), primary diseases, and history of basic diseases) and other variables like smoking, resident area, type of dialysis, dialysis time, and times of dialysate change were registered. Furthermore, most inflammatory indexes, alanine aminotransferase (ALT), albumin (ALB), aspartate aminotransferase (AST), white blood cell (WBC) count, neutrophil (NE), platelet (PLT) count, red blood cell (RBC), hemoglobin (Hb), hematocrit (HCT), blood urea nitrogen (BUN), serum creatine (sCr), serum glucose (GLU), and so on were all carefully recorded.

According to the diagnostic criteria of PDRP, these patients were separated into the PDRP group (n=25) and the control group (n=45). The PDRP group were diagnosed by following two of the three conditions: 1) agreeing to the clinical features of peritonitis, that is, bellyache and/or turbidity in dialysate; 2) white blood cell count >100/μl (with more than 2 h of abdominal retention time), and proportion of polymorphonuclear >50%; 3) a positive result was obtained in the microbial culture of the dialysate. Based on the clinical indexes of the patients, we analyzed the potential risk factors of PDRP by comparing the difference of the indexes between the two groups.

### Sample preparation and performance test

Dialysis effluent samples from the patients undergoing PD were collected for pathogen identification by mNGS and microbial culture, including 35 PDRP and 3 PD patients without peritonitis. The clinical microbial culture was performed in the First Hospital of Jilin University. The performance of these two methods, represented by sensitivity, specificity, positive predictive value (PPV), and negative predictive value (NPV), was compared. 2 × 2 contingency tables were adopted to calculated them: sensitivity = TP/(TP+FN); specificity = TN/(TN+FP); PPV = TP/(TP+FP); NPV = TN/(TN+FN) (TP: true positive; FN: false negative; TN: true negative; FP: false positive).

### mNGS workflow

The process of mNGS, including DNA extraction, library construction, sequencing procedure, bioinformatic analysis, and data interpretation, was performed by the Genoxor Medical Technology (Shanghai, China). DNA was extracted directly from dialysis effluent with the TIANamp Micro DNA Kit (DP316, Tiangen Biotech, Beijing, China) according to the manufacturer’s standard protocols. Qubit 3.0 (Thermo Fisher Scientific, Waltham, USA) was used for DNA concentration and purity detection. After being fragmented into 200 bp–300 bp, DNA underwent end repair, adapter ligation, and PCR amplification to produce the DNA libraries. The concentration and quality of DNA libraries were measured by Qubit 3.0 and Agilent 2100 system (Agilent Technologies, Santa Clara, USA). The qualified DNA libraries were put into sequencing on the NextSeq™ 550Dx platform in SE-75 type.

### Bioinformatic analysis

The raw sequencing data initially underwent a quality control process by removing low-quality reads and tails and by connector sequencing via the Trimmomatic v0.36 software (20). The obtained clean reads were filtered to remove the human genome by aligning to the human reference genome GRCh37 with the short-read alignment tool Bowtie v2.2.629 (21). The remaining microbial reads data are deposited in the NCBI Sequence Read Archive database under the access number of PRJNA1081608. A genome database in NCBI established using 51,543 genomes of approximately 27,000 species (ftp://ftp.ncbi.nlm.nih.gov/genomes/) was referenced to identify the sequences of microbial species (22). The percentage relative abundance of each species was normalized via a method called reads per kilobase of transcript per million mapped reads (RPKM) as described previously (23). RPKM was computed with a formula: gene reads/[the total mapped reads (millions) × genome length (kb)].

### Gut microbiota analysis

Anal swabs were obtained from 35 PDRP patients and underwent mNGS workflow for further gut microbiome analysis. 35 months of dialysis time were taken as the node; patients with a dialysis time less than or equal to 35 months were divided into the DT35 group (n=20), whereas patients with more than 35 months of dialysis time were assigned to the DT36 group (n=15). According to the discrepancy in times of dialysate change, the PDRP patients were divided into TDC4 (times of dialysate change per day ≤4, n=14) and TDC5 (times of dialysate change per day >4, n=6) groups. In the PDRP patients, the putative pathogens and causative agents were judged based on a comprehensive analysis of clinical manifestations, clinical epidemiology, radiologic results, pathogen detection, and the treatment outcome of antibiotic therapy. Based on the causative pathogens causing infection in PDRP patients, we divided them into gram-positive (G+, n=9) and gram-negative (G−, n=10) groups to analyze their gut microbiota.

The online system called statistical analysis and visualization of metagenomic sequencing (SAV-mNGS) (http://192.168.1.229:3838/SAV-mNGS/) that was developed and maintained by Genoxor Medical Science and Technology Inc. (Shanghai, China) was applied for microbiota analysis. SAV-mNGS, an R Shiny application for researchers and clinicians to analyze and visualize clinical metagenomic sequencing data, was applied for 1) global visualization of sampling effort and distribution of dominant taxa among groups or individual samples at the species levels; 2) data filtering to reduce the abnormal samples or taxa, and data normalization; 3) statistical analysis and visualization of α diversity and β diversity; 4) principal component analysis (PCA) and analysis of similarity (ANOSIM) of β diversity on Bray–Curtis or UniFrac distance and visualization; 5) prediction of microbial biomarkers specific to individual groups with various statistical and machine learning approaches; 6) linear discriminant analysis effect-size (LEfSe) analysis (LDA >2) compared two groups of microbiota to screen for biomarkers with statistical differences; 7) assessment of the clinical validity of selected biomarkers by receiver operating characteristic (ROC) curve and area under the curve (AUC). Setting 25% of the samples randomly selected from the sample population as the validation set to verify the classifier, fivefold cross-validation was adopted. α diversity (within community diversity) and β diversity (diversity between a group of samples) form the overall diversity in environmental communities. PCA was adopted to assess the dispersion degree of samples in different groups. ANOSIM was performed to determine the inter-group and intra-group microbiome differences between the two groups.

### Statistical analysis

Mean represented numerical values conforming to the normal distribution and standard deviation (M ± SD), and the others were represented by median (first quartile) [M (QR)]. Logical regression analysis was performed to assess the independent risk factor of PDRP. Chi-square testing was performed to analyze the data between the two groups. The relationship between the probability of non-peritonitis in patients and changes in number of dialysate changes and dialysis time was determined using the Kaplan–Meier survival curve. Statistical analyses and visualization were performed using GraphPad 8.0.2 (GraphPad Prism Software Inc., San Diego, CA). A *P* value of <0.05 was considered significant.

## Results

### Patient baseline

A total of 70 patients who received PD in the First Hospital of Jilin University were included in this study, including 31 men and 39 women, ranging from 24 to 74. According to the diagnostic criteria of PDRP, these patients were separated into the PDRP group (n=25) and the control group (n=45). The baseline information is listed in **Table 1**. The mean patients’ age was 51.8 years old in the PDRP group and 50.51 years old in the control group.

**Table 1:** Patient characteristics.

	PDRP group (n=25)	Control (n=45)	x²/t	P
Sex (male/female)	12/13	19/26	0.2174^a^	0.641
Age (years, m ± SD)	51.8 ± 10.7	50.51 ± 12.7	0.4294^b^	0.669
BMI (kg/m^2^)	24.3 ± 3.9	22.6 ± 3.6	1.764^b^	0.0824
Primary diseases (%)				
Unclear	9 (36%)	18 (40%)	0.1085^a^	0.7418
Diabetic nephropathy	5 (20%)	9 (20%)	0.000^a^	1
Hypertensive nephropathy	3 (12%)	6 (13.3%)	0.02550^a^	0.8731
Primary glomerular disease and renal atrophy	8 (32%)	12 (26.7%)	0.05983^a^	0.8068
History of basic diseases (%)				
Have basic diseases	22 (88%)	42 (93.3%)	0.5833^a^	0.445
Hypertension	22 (88%)	40 (88.88%)	0.01254^a^	0.9108
Diabetes mellitus	8 (32%)	11 (24.4%)	0.4639^a^	0.4958

PDRP, peritoneal dialysis-related peritonitis; BMI, body mass index; DM, diabetes mellitus.

^a^refers to x², ^b^refers to t; numerical values conforming to normal distribution were represented by mean ± SD, and the others were represented by median (first quartile) [M (QR)].

In 36% of PDRP patients, their primary disease information was absent, and the rest suffered from diabetic nephropathy (20%), hypertensive nephropathy (12%), and primary glomerular diseases and renal atrophy (32%). In the control group, the primary diseases also included diabetic nephropathy (20%), hypertensive nephropathy (13.3%), and primary glomerular diseases (26.7%), but the information on primary diseases was absent in 40% of them. In addition, 88% (PDRP) and 93.3% (control) of the patients have primary diseases, like hypertension (88% and 88.88%) and diabetes mellitus (32% and 24.4%). No significant difference (*P*>0.05) was noted between the two groups in sex, age, BMI, primary diseases, and the history of basic diseases (**Table 1**).

### Analysis of risk factors for PDRP

To assess the risk factors for PDRP, the main variables in the two groups were analyzed, such as smoking, residential place, diabetes mellitus, and dialysis types (**Table 2**). It indicates that significant difference was found in dialysis time (*P*=0.0186) between PDRP and control groups. The median dialysis time of PDRP patients was 36 months, whereas it was 18 months in the control group. Meanwhile, times of dialysate change (*P*=0.0095) was also significantly different between the PDRP and control groups. Logical regression analysis indicated that times of dialysate change was the independent risk factor of PDRP (*P*=0.046). Along with the prolonged dialysis time and increased dialysate change times, the probability of non-peritonitis was decreased; that is, the incidence of peritonitis was elevated (**Supplementary Figure 1**). However, the other factors, including PD machine application and laboratory results like BUN, sCr, and GLU were not significantly different between the two groups (*P*>0.05).

**Table 2:** Risk factors of PD-related peritonitis.

	PDRP group (n=25)	Control group (n=45)	x²/t/z	P
Smoking (yes or no)	8/17	8/37	1.844^a^	0.1745
Diabetes mellitus (yes/no)	8/17	11/34	0.01445^a^	0.9043
Residential place (rural/urban)	7/18	13/32	0.006222^a^	0.9371
Dialysis types (PD/PD+HD)	21/4	43/2	2.738^a^	0.098
Peritoneal dialysis machine (yes/no)	3/22	8/37	0.4051^a^	0.5245
Dialysis time [M(QR)]	36 (18)	18 (11)	371.5	0.0186
Times of dialysate change [M (QR)]	4 (4)	4 (3)	387.5	0.0095
BUN	17.6 ± 5.3	16.6 ± 5.0	0.7773^b^	0.4397
sCr	779.6 (634.9)	695.5 (590.9)	475	0.2885
GLU [M(QR)]	5.32 (4.93)	5.44 (5.09)	525.5	0.7637
ALT [M(QR)]	13.8 (11.725)	12.7 (9.7)	493	0.3986
AST [M(QR)]	18.3 (13.6)	17.4 (13.9)	550.5	0.8863
ALB	34.28 ± 3.57	33.43 ± 5.29	0.7183^b^	0.475
TC	4.7 ± 1.28	4.34 ± 1.84	0.4436^b^	0.6588
TG [M(QR)]	1.58 (1.02)	1.4 (1.04)	477.5	0.3011
HDL [M(QR)]	1.16 (0.98)	1.07 (0.9)	479.5	0.3127
LDL	2.82 ± 1.02	2.51 ± 1.14	0.01426^b^	0.9887
WBC *10^9	7.57 ± 3	6.87 ± 1.75	1.708^b^	0.0923
NE *10^9	5.71 (2.4)	4.6 (1.45)	446.5	0.157
LY% [M(QR)]	0.2 (0.18)	0.2 (0.17)	530	0.6939
PLT	226 ± 78.6	195 ± 70.89	0.8844^b^	0.3796
RBC	3.77 ± 0.67	3.53 ± 0.79	1.292^b^	0.2008
Hb [M(QR)]	113 (96)	107 (95)	472	0.2706
HCT	0.34 ± 0.06	0.32 ± 0.06	1.765^b^	0.0821

PDRP, peritoneal dialysis-related peritonitis; BMI, body mass index; HD, hemodialysis; BUN, urea nitrogen; sCr, serum creatinine; GLU, glucose; ALT, alanine aminotransferase; AST, aspartate aminotransferase; ALB, albumin; TC, total cholesterol; TG, triglyceride; HDL, high-density lipoprotein; LDL, low density lipoprotein; WBC, white blood cell count; NE, neutrophil; LY, lymphocyte; PLT, platelet count; RBC, red blood cell; Hb, hemoglobin; HCT, hematocrit.

^a^refers to x², ^b^refers to t, other with no annotations refers to u; numerical values not conforming to normal distribution were represented by median (first quartile).

### Comparison of detection performance between mNGS and microbial culture

**Figure 1:**
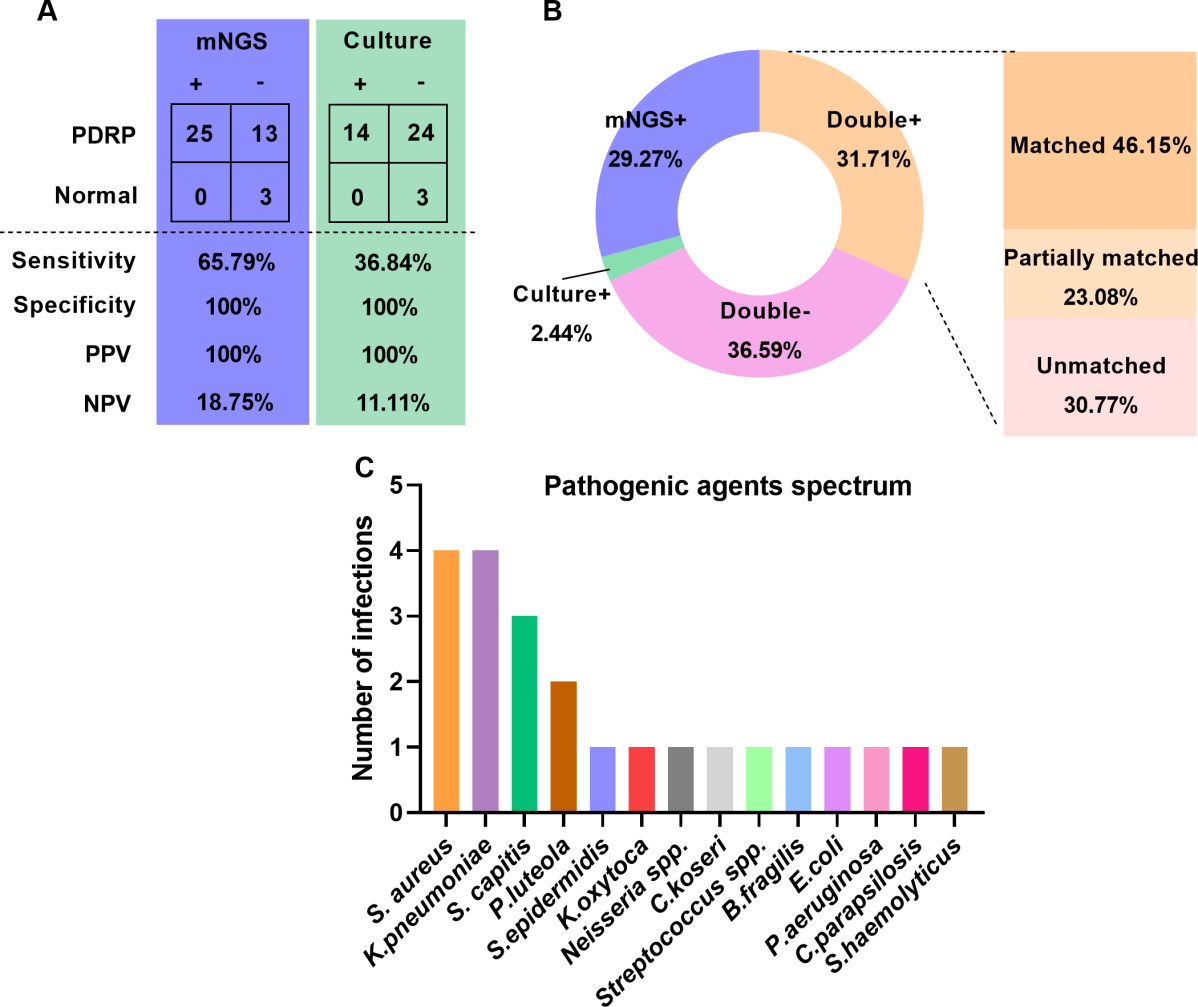
Comparison of detection performance between mNGS and microbial culture. **(A)** Comparison of sensitivity and specificity between mNGS and microbial culture. **(B)** Comparison of positive and negative results and concordance by mNGS and microbial culture. **(C)** Causative agents in patients with PDRP.

There were 41 patients (38 with PDRP, 3 without PDRP) who were recruited to assess the detection performance of mNGS and culture in diagnosing PDRP. Taking the clinical diagnosis as the gold standard, the sensitivities of mNGS and microbial culture were 65.79% and 36.84%, respectively, and the specificity of mNGS and microbial culture all reached 100% (**Figure 1A**). PPV and NPV of the mNGS method were 100% and 18.75%, separately, and those of microbial culture were 100% and 11.11%, respectively (**Figure 1A**). Furthermore, we compared the consistency of mNGS and microbial culture results. As shown in **Figure 1B**, in general, the positive rate of mNGS detection was higher than that of culture (60.69% vs. 34.15%). Among the 13 patients (31.71%) who were positive in both mNGS and culture methods, we further analyzed the matching of the two detection results. Among them, six cases (46.15%) were completely matched (the pathogens detected by mNGS and culture were completely matched); three cases (23.08%) were partially matched (at least one microorganism overlapped between mNGS and culture); and four cases (30.77%) were mismatched (there was no pathogen overlap between the results of mNGS and culture tests).

In the 38 PDRP patients, one or more causative pathogens were identified in 22 (57.89%) cases, according to a comprehensive analysis of clinical data, including clinical manifestations and pathogen detection findings. In the remaining 16 patients, the pathogenic agent was not determined. The spectrum of pathogenic agents was shown in **Figure 1C**, demonstrating that *Staphylococcus aureus* and *Klebsiella pneumoniae* (four times) were the most frequently pathogenic microorganisms in the PDRP patients, followed by *Staphylococcus capitis* (three times) and *Pseudomonas luteola* (two times).

### Gut microbiota analysis in peritoneal dialysis patients

**Figure 2:**
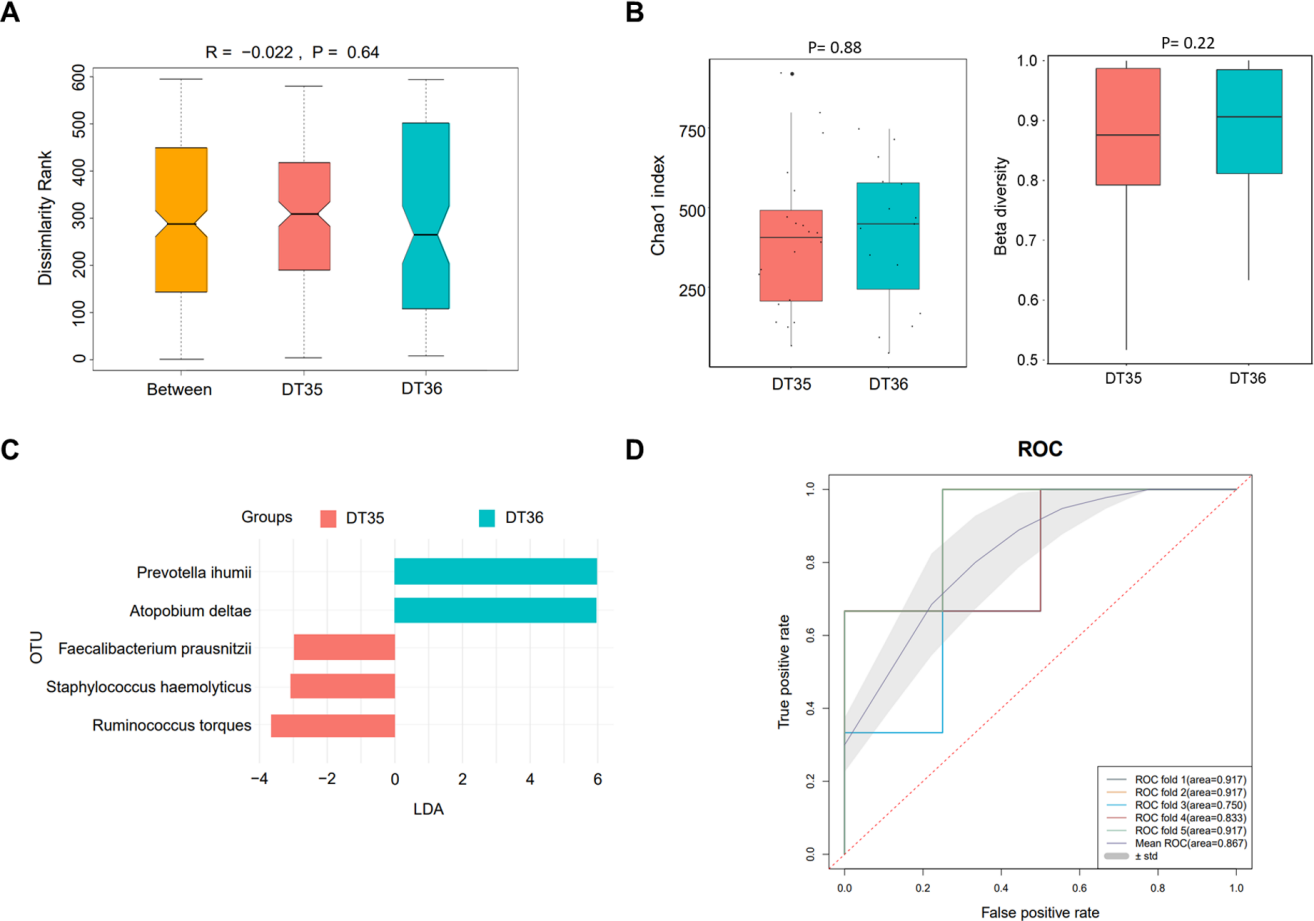
Microbiome analysis in PDRP patients with different dialysis time (less than or equal to 35 months and more than 35 months). **(A)** Analysis of similarity of β diversity to assess the inter-group and intra-group microbiome differences between the two groups. **(B)** Comparison of α diversity and β diversity of the microbiota between the two groups. **(C)** LDA revealed the species with significant difference in the two groups. **(D)** ROC curve used to evaluate the clinical validity of species with an LDA value equal or more than two.

The above results revealed that dialysis time and times of dialysate change were the risk factors of PDRP. Herein, we grouped the patients based on these two factors to analyze their gut microbiomes to explore the difference in their etiological characteristic. Between the DT35 (n=20) and DT36 (n=15) groups, the R value (−0.022) of ANOSIM indicated a larger difference in intra-group than in inter-group, but the difference was not significant (*P*=0.74) (**Figure 2A**). No significant difference was found in α diversity (Chao1 index, P=0.88) and β diversity (P=0.22) of the microbiota between the two groups of patients with different dialysis times (**Figure 2B**). LEfSe analyses found five microbial species significantly different between the DT35 and DT36 groups, with *Prevotella ihumii* and *Atopobium deltae* showing the highest linear discriminant analysis value (LDA=6) (**Figure 2C**). The ROC curve suggested a high sensitivity and specificity of the five microbial species in diagnosing the PDRP, with the mean AUC of 0.867 (**Figure 2D**).

**Figure 3:**
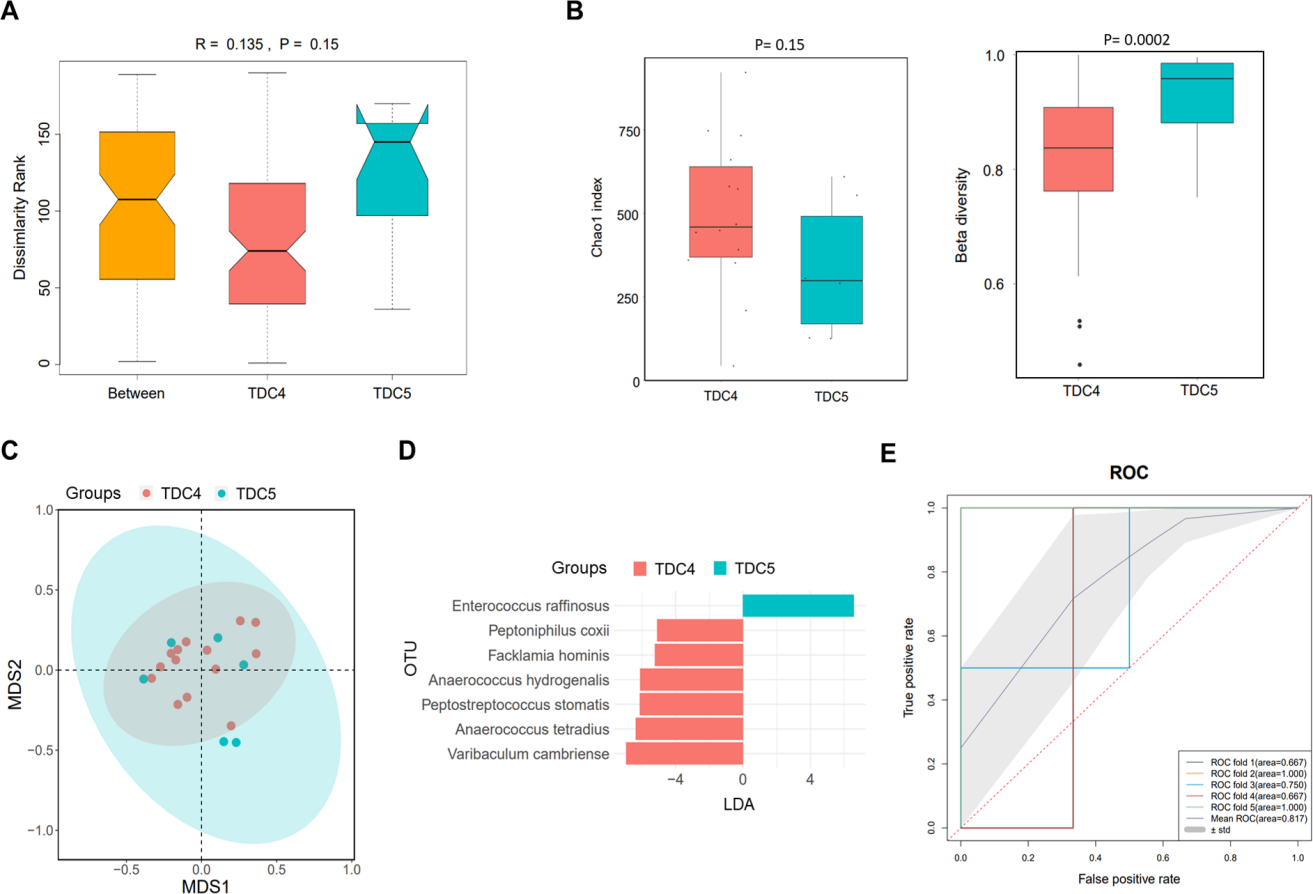
Microbiome analysis in PDRP patients with different times of dialysate change (less than or equal to four times and more than four times). **(A)** Analysis of similarity of β diversity to assess the inter-group and intra-group microbiome differences between the two groups. **(B)** Comparison of α diversity and β diversity of the microbiota between the two groups. **(C)** PCA was performed to assess the average microbial composition diversity of the two groups. **(D)** LDA revealed the species with significant difference in the two groups. **(E)** ROC curve used to evaluate the clinical validity of species with a LDA value equal or more than two.

According to the discrepancy in times of dialysate change, the PDRP patients were divided into TDC4 (n=14) and TDC5 (n=6) groups. As shown in the ANOSIM result, an R value of 0.135 was acquired, demonstrating that inter-group difference was higher than intra-group difference, but no statistic difference was observed (*P*=0.15) (**Figure 3A**). We did not find significant difference in α diversity (Chao1 index) between the TDC4 and TDC5 groups (*P*=0.15) but noted that the β diversity in the TDC4 group was significantly lower than in the TDC5 group (*P*=0.0002) (**Figure 3B**). The result of PCA manifested clear separation in samples between TDC4 and TDC5 groups (**Figure 3C**). The LEfSe result found seven microbial species with significant difference between the two groups, and *Enterococcus raffinosus* was the most different species between the two groups (**Figure 3D**). In addition, the ROC revealed the mean AUC of 0.817 (**Figure 3E**), highlighting the reliability of this model in forecasting PDRP.

**Figure 4:**
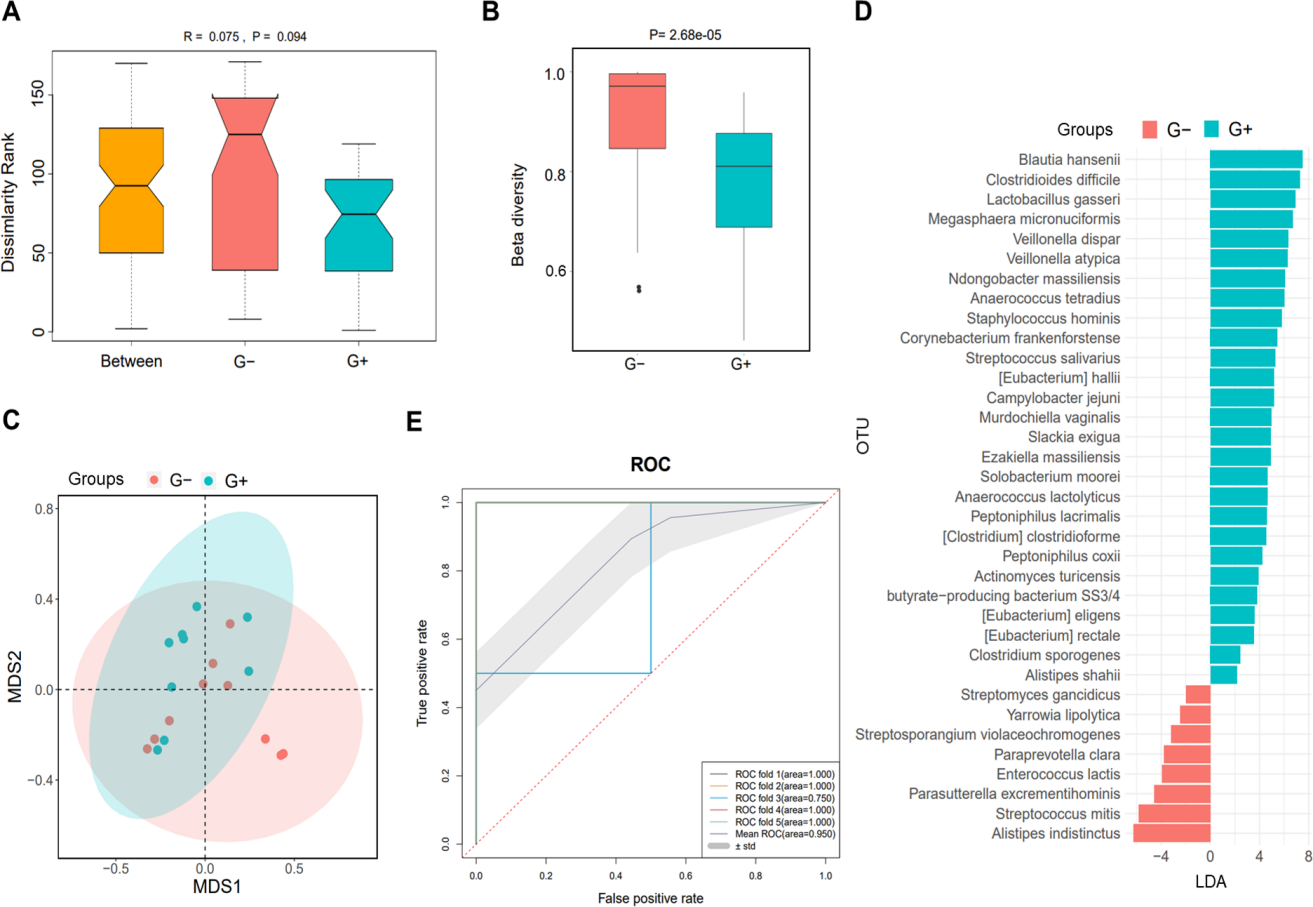
Microbiome analysis in PDRP patients infected with gram-positive (G+) bacterial and gram-negative (G−) bacteria. **(A)** Analysis of similarity of β diversity to assess the inter-group and intra-group microbiome differences between the two groups. **(B)** Comparison of α diversity and β diversity of the microbiota between the two groups. **(C)** PCA was performed to assess the average microbial composition diversity of the two groups. **(D)** LDA revealed the species with significant difference in the two groups. **(E)** ROC curve used to evaluate the clinical validity of species with an LDA value equal or more than two.

Based on the causative pathogen leading to infection in PDRP patients, we divided them into G+ (n=10) and G− (n=9) groups to analyze their gut microbiota. The result of ANOSIM indicated that the inter-group difference in the microbial community was slightly larger than the intra-group difference, with an R-value of 0.075 and a *P*-value of 0.094 (**Figure 4A**). β diversity in the patients with G+ bacterial infection was clearly lower than with G− bacterial infection (*P*= 2.68e−05, **Figure 4B**). PCA showed that samples in G+ and G− groups were distinctly separated, implying that the microbiome in the two populations was significantly distinct (**Figure 4C**). Meanwhile, various species were identified with significant differences between G+ and G− groups, such as *Blautia hansenii* (LDA=7.5) and *Clostridioides difficile* (LDA=7.3) (**Figure 4D**). The ROC demonstrated that this predictive model was excellent, with the mean AUC value reaching 0.950 (**Figure 4E**).

## Discussion and conclusion

In this study, we proved that dialysis time and the times of dialysate change were credible risk factors of PDRP. For pathogen identification, mNGS performed outstandingly in sensitivity compared with the traditional culture method and was a promising tool in the etiological diagnosis of PDRP. Furthermore, gut microbiome analyses demonstrated that β diversity was significantly different between patients with fewer times of dialysate change (≤4) and more (>5), as well as between patients with G+ bacterial and G− bacterial infection. These findings represent the global microbiome landscape in PDRP patients, providing an essential theoretical basis for specific nursing strategies to reduce the incidence of peritonitis.

Several frequent risk factors lead to PDRP, including patients’ self-factors, such as obesity, diabetes mellitus, malnutrition, and the weakened function of the intestinal mucosa barrier (10, 24). Iatrogenic factors include the implantation of a dialysis catheter, catheter-related factors, and non-aseptic operation when changing the dialysate (25, 26). Herein, our data manifested that longer dialysis time and more times of dialysate change meant a higher incidence rate of peritonitis in PD patients (**Table 1**). This may be because prolonged dialysis disrupts the peritoneal defense mechanism of patients. The repeated exchanges of dialysis fluid change the physiological environment of the abdominal cavity, including the loss of the homeostatic and anti-inflammatory potential characteristics of peritoneal macrophages and the reduction of membrane complement activity. These changes have an adverse effect on the specific and non-specific immune functions of patients, making the peritoneum susceptible to bacteria (27–29). In addition, an increase in the time of dialysate change may be accompanied by an increased risk of catheter-related infections. It is known that catheter-related infections in peritoneal dialysis (PD) are important risk factors for catheter loss and peritonitis. For example, if medical staff do not follow aseptic operation principles, bacteria in the mouth and nose can contaminate pipelines and connectors through the air and hands when changing dialysis fluid (30, 31). Therefore, careful operation when implanting the dialysis catheter to avoid mechanical injury of the peritoneum is urgently needed, as well as fixing the catheter and strict aseptic operation, which are the critical points in preventing PDRP.

A recent study concerning PDRP patients reports that the positive rate of the mNGS test is higher than that of microbial culture for pathogenic microorganism identification but lacks any statistical significance (32). Our results showed that mNGS produced higher sensitivity (65.79%) than microbial culture (36.84%) (**Figure 1A**), displaying a better etiological diagnostic value in evaluating PDRP than the culture method. In the samples with double-positive results in these two methods, their concordance rate is 46.15%, which is attributed to the fact that mNGS detected more species than microbial culture. The poor consistency may also be due to some potential sources of errors. These include incorrect databases in the mNGS test and parameter settings that may lead to errors in species identification or abundance. mNGS relies on the quality and efficiency of nucleic acid extraction. The detection results are influenced by factors such as sample collection, preservation, and extraction methods. mNGS can detect almost all microbial nucleic acid sequences in a sample. However, microbial culture may not be able to detect some fastidious bacteria, slow-growing microorganisms, or microorganisms that cannot be cultured. mNGS has higher detection sensitivity than the culture method and can detect low-abundance microorganisms. In addition, the use of antibiotics may also affect the growth of microorganisms and detection results. Collecting samples for detection after antibiotic treatment may lead to inconsistent results of the two tests. Nie and colleagues proved that the positivity rate using mNGS was 92.3%, whereas the positivity rate of microbial culture was 38.5% in the antibiotic-use group (15). It suggests that the detection efficiency of mNGS is higher and is free of antibiotic usage. It follows that mNGS technology is promising to be widely applied in diagnosing PDRP and other infectious diseases.

Generally, most PDRP episodes were caused by gram-positive organisms (57%), as cocci are the dominant etiological agents of peritonitis, with staphylococci being the most common microbial agents (32, 33). The organism most often cultured was coagulase-negative *Staphylococcus* (30%), followed by *S. aureus*, *Streptococcus*, and *Enterococcus* species; in 11% of all peritonitis cases, gram-negative organisms such as *Pseudomonas* species and *Escherichia coli* were found (34). Our spectrum of pathogenic agents of PDRP showed that *Staphylococcus aureus* and *Klebsiella pneumoniae* (four cases) were the most frequent pathogens, followed by *Staphylococcus capitis* (three cases). Among these causative pathogens in our study, *Staphylococcus aureus* in all four cases were solely detected by mNGS. It has been reported that *Staphylococcus aureus*-related peritonitis is associated with more severe episodes, a higher method failure rate, and a higher mortality rate in PD patients (35, 36). Therefore, mNGS contributes more to identifying the etiological agents of PDRP, which is crucial for the timely treatment of peritonitis in PD patients.

Most endogenous microorganisms derive from the enteric canal, and the migration and invasion of intestinal flora are the principal origins of pathogens causing PDRP (19, 37). A previous analysis of fecal community diversity showed an obvious change in the microbial structure of PDRP patients when compared with the normal PD patients (38). Herein, we demonstrated that gut microbial community distribution was significantly altered along with the increased times of dialysate change (**Figure 3B**) in PDRP patients. That is, dialysate change operation might be the primary cause of gut microbiota alteration in PD patients. Accompanied by altered gut microbiota, the accumulation of detrimental metabolite increased the risk of endogenous infection in PD patients. As Zhang et al. have reported that gut microbial metabolite trimethylamine-N-oxide increases peritoneal inflammation and peritonitis risk in PD patients (19). Even gram-positive bacteria are the common causative pathogens in PDRP, a recent 10-year study reveals that the proportion of gram-negative bacterial peritonitis increased, with *E. coli* being the leading cause of gram-negative bacterial peritonitis, and the prognosis of gram-negative bacterial peritonitis is worse (39). Our data found no significant difference in the proportion of G+ (10 cases) and G− (9 cases) bacterial peritonitis. Intra-abdominal infections caused by gram-negative bacteria are generally associated with endogenous factors such as the migration of intestinal flora. Nevertheless, the gram-positive bacteria-induced intra-abdominal infections result from improper wound care. Therefore, comparison of gut microbiota between the two populations will benefit to improve the recognition on the pathogenic microorganisms leading to peritonitis in PD patients. Herein, we have initially discovered that patients with Gram-negative bacterial infection showed higher β diversity than those infected with Gram-positive bacteria (**Figure 4B**). A study involved in *E. coli* peritonitis revealed that pathways involving in the digestive system, energy metabolism, folding, sorting and degradation, and glycan biosynthesis and metabolism were significantly reduced in *E. coli* peritonitis patients (40). In some extent, it supports the viewpoint that gram-negative bacteria are responsible for the worse gut microenvironment. Given this, we recognize that the infection induced by gram-negative bacteria could be avoided by improving the intestinal micro-ecology. The AUC values in ROC curve analysis were all greater than 8, indicating that the biomarkers obtained from LEfSe analysis have good classification performance. However, since we only used 25% of the total sample for the test set, this may lead to an overestimation of the classifier’s performance. In the future research, it is necessary to establish an external dataset to validate the model.

There are several limitations to the study. First, we included a small sample size in our study, which affected the generality of the results. Moreover, the samples were only from one center in the same hospital, so the samples may be biased and do not fully represent the overall PD and PDRP patients. Second, in the comparison of pathogen detection performance, we only compared mNGS and culture methods, which is not comprehensive. In fact, Gram staining, biochemical identification, serological testing, and polymerase chain reaction (PCR) techniques are also widely used. In the future, we will recruit participants from more regions and different hospitals to improve the reliability and generalization of the study results. In addition, further prospective studies or setting up validation cohorts will help to confirm these analysis results.

In summary, dialysis time and times of dialysate change are the risk factors of PDRP, and times of dialysate change is the independent risk factor. More times of dialysate change is accompanied by altered gut microbial community structure and distribution. Gram-negative bacteria peritonitis may be derived from the worsening gut microenvironment. Meanwhile, mNGS performed better than microbial culture in pathogen identification, with higher sensitivity and a broader spectrum of pathogen, and is expected to be a novel method in rapid etiological diagnosis of PDRP.

## Data Availability

The datasets presented in this study can be found in online repositories. The names of the repository/repositories and accession number(s) can be found in the article/**Supplementary Material**.
