# Supplementary material for: Risk factor assessment and microbiome analysis in peritoneal dialysis-related peritonitis reveal etiological characteristics
Source: Front Immunol. 2024 Nov 14;15:1443468. doi: 10.3389/fimmu.2024.1443468 (PMC11602453; doi:10.3389/fimmu.2024.1443468)
Supplement: Supplementary file 1 [file DataSheet1.pdf]

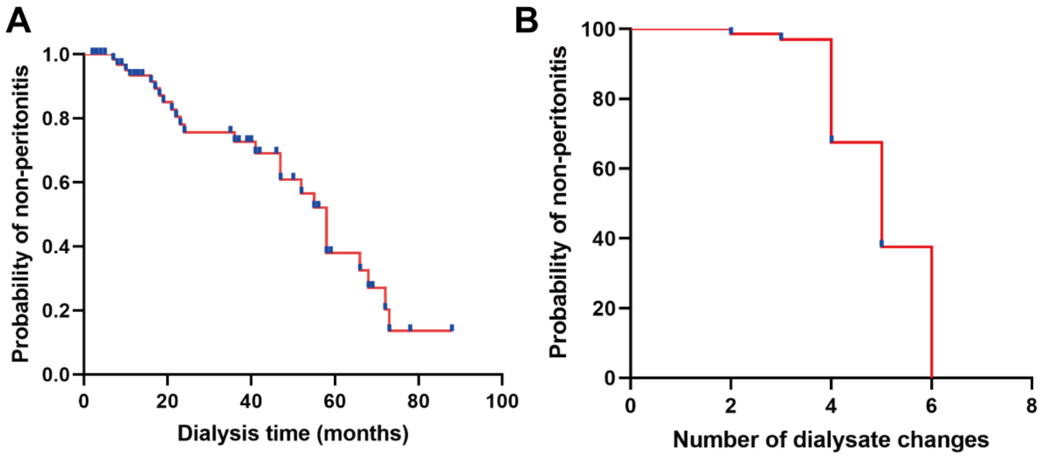

Supplementary Figure 1 Survival curve of peritonitis-free in PD patients. (A) Survival curve of peritonitis-free in PD patients with different dialysis time; (B) Survival curve of peritonitis-free in PD patients with different frequency of dialysate change.
